# Prolonged AT_1_R activation induces Ca_V_1.2 channel internalization in rat cardiomyocytes

**DOI:** 10.1038/s41598-017-10474-z

**Published:** 2017-08-31

**Authors:** Tamara Hermosilla, Matías Encina, Danna Morales, Cristian Moreno, Carolina Conejeros, Hilda M. Alfaro-Valdés, Felipe Lagos-Meza, Felipe Simon, Christophe Altier, Diego Varela

**Affiliations:** 1 Programa de Fisiopatología, Instituto de Ciencias Biomédicas, Facultad de Medicina, Universidad de Chile, Santiago, 8380453 Chile; 2 Departamento de Ciencias Biológicas, Facultad de Ciencias Biológicas and Facultad de Medicina, Universidad Andrés Bello, Avenida Republica 239, Santiago, 8370146 Chile; 3 Department of Physiology and Pharmacology and Snyder Institute for Chronic Diseases, University of Calgary, Calgary, T2N 4N1 Canada; 4 Millennium Institute on Immunology and Immunotherapy, Santiago, 8331150 Chile; 5 Millennium Nucleus of Ion Channels-Associated Diseases (MiNICAD), Santiago, Chile

## Abstract

The cardiac L-type calcium channel is a multi-subunit complex that requires co-assembling of the pore-forming subunit Ca_V_1.2 with auxiliary subunits Ca_V_α_2_δ and Ca_V_β. Its traffic has been shown to be controlled by these subunits and by the activation of various G-protein coupled receptors (GPCR). Here, we explore the consequences of the prolonged activation of angiotensin receptor type 1 (AT_1_R) over Ca_V_1.2 channel trafficking. Bioluminescence Resonance Energy Transfer (BRET) assay between β-arrestin and L-type channels in angiotensin II-stimulated cells was used to assess the functional consequence of AT_1_R activation, while immunofluorescence of adult rat cardiomyocytes revealed the effects of GPCR activation on Ca_V_1.2 trafficking. Angiotensin II exposure results in β-arrestin_1_ recruitment to the channel complex and an apparent loss of Ca_V_1.2 immunostaining at the T-tubules. Accordingly, angiotensin II stimulation causes a decrease in L-type current, Ca^2+^ transients and myocyte contractility, together with a faster repolarization phase of action potentials. Our results demonstrate that prolonged AT_1_R activation induces β-arrestin_1_ recruitment and the subsequent internalization of Ca_V_1.2 channels with a half-dose of AngII on the order of 100 nM, suggesting that this effect depends on local renin-angiotensin system. This novel AT_1_R-dependent Ca_V_1.2-trafficking modulation likely contributes to angiotensin II-mediated cardiac remodeling.

## Introduction

In mammals, the renin-angiotensin system (RAS) is one of the key factors in the regulation of blood pressure, electrolyte balance and cardiac function. Renin catalyzes the conversion of the 14 amino acid propeptide angiotensinogen into angiotensin I (AngI), which in turn is cleaved by the angiotensin-converting enzyme (ACE) to produce angiotensin II (AngII)^1^. AngII participates in a series of intracellular signaling cascades via activation of its receptors AT_1_ and AT_2_, which belong to the superfamily of seven-transmembrane domain G-protein coupled receptors (GPCR)^2^.

Most of the classical actions associated with AngII are dependent on the activation of the AT_1_ receptor (AT_1_R)^3^. Binding of AngII results in rapid phosphorylation of the receptor that facilitates β-arrestin recruitment induced desensitization and internalization of AT_1_R^4, 5^. Thus, activated AT_1_R is phosphorylated by GPCR kinases (GRKs) and subsequently β-arrestin is recruited to the receptor. This receptor/β-arrestin interaction leads to receptor desensitization, ending the G protein-dependent signaling by interfering with the coupling between the G protein and its receptor. Furthermore, β-arrestin can also bind to components of the clathrin coat, causing internalization of activated receptors and target to degradation pathways or recycled back to the cell surface^6, 7^.

Several recent studies have demonstrated the existence of macromolecular complexes involving GPCRs and various ion channels. For example, the dopamine D1 receptor regulates the NMDA receptor through a direct interaction^6^. AT_1_R co-immunoprecipitates with the non-selective cation channel TRPV4^7^ and with K_V_4.3, which is responsible for the fast transient outward current (I_to_) in cardiac myocytes^8^. Likewise, it has been shown that voltage dependent calcium channels (VDCC) interact with various GPCRs. For instance, N-type Ca^2+^ channels interact with ORL1^9^, GABAb^10^ and the dopamine D1^11^ and D2^12^ receptors. Interestingly, most of these interactions lead to endocytosis of the channel following activation of the respective receptor^8, 9, 11, 13^.

In general, VDCCs are protein complexes involving at least three subunits: a Ca_V_α_1_ subunit that forms the channel pore, and Ca_V_α_2_δ and Ca_V_β accessory subunits, responsible for trafficking of the channel complex^14^ to the plasma membrane. In cardiomyocytes, LTCC clusters, with specific subcellular localization, have been associated with specific functions. Thus, LTCC localized at the T-tubules are mainly responsible for excitation-contraction coupling by its association with ryanodine receptor 2 (RyR2) channels, while LTCC localized at the sarcolemma are implicated in signaling to the nucleus and regulation of gene transcription^15^.

Several studies have convincingly shown that AngII modulates L-type currents. However, this modulation appears to depend on the cell type and on the species studied. For example, AngII induces an increase of L-type currents in smooth muscle cells^16^ by a PI3Kdependent mechanism^17, 18^, while in neurons^19, 20^ and in glomerular cells, a decrease in these currents has been observed via a Pertussis toxin sensitive G-protein pathway^21^.

In cardiomyocytes, the effect on this current after AngII application has been shown to be dependent on the species and the patch-clamp configuration used. In hamster^22^ and guinea pig^19^ the L-type current is increased, while in rabbit^23^ and cat^24^ no change is observed in cells studied with conventional whole-cell (WC) recordings, in contrast, perforated patch experiments show a potentiation of the L-type current. Finally, in rat^25^ and human^26^ a decrease of the L-type current has been reported using the conventional WC configuration, while the use of perforated patch configuration in newborn rat cardiomyocytes shows that the degree of L-type current inhibition depend on the speed of inactivation of L-type currents^27^. This evidence together with a growing number of reports, establishes a clear link between VDCC and GPCRs, suggesting the existence of a functional association between L-type calcium channels and AT_1_R.

In this work, we demonstrate that AngII stimulation of AT_1_R causes β-arrestin_1_ (but not β-arrestin_2_) recruitment of the L-type calcium channel complex. From a functional stand point, AT_1_R activation induces internalization of the T-tubule Ca_V_1.2 population after prolonged exposure to AngII, leading to ~60% reduction of endogenous L-type calcium currents, diminished calcium transient amplitude, and reduced action potential duration.

## Methods

### Constructs

cDNA encoding the angiotensin receptor 1, AT_1_R (NM_000685), was obtained from the University of Missouri-Rolla cDNA resource center. cDNAs for calcium channel subunits (GenBank™ accession numbers: X15539 (Ca_V_1.2) and AF286488 (Ca_V_α_2_δ_1_) were kindly provided by Dr. T. Snutch. Ca_V_β_2b_ channel subunit (AF423193) was cloned as described in Moreno *et al*.^28^. The HA epitope inserted into the extracellular S5-H5 loop of domain II of Ca_V_1.2 for the HA-tagged Ca_V_1.2 construct is described previously^29^. Ca_V_1.2-YFP and Ca_V_β_1b_-YFP were PCR amplified and subcloned into the N1-YFP vector (Clontech) between the NheI and BamHI or EcoRI and BamHI restriction sites respectively. β-arrestin-Rluc constructs were kindly provided by Dr. R. Ramachandran and AT_1_R-YFP construct was kindly provided by Dr. LM Luttrell.

### AD-293 culture and transfection

Tissue culture of AD-293 cells (Agilent) was performed as recommended by the distributor. Transfection solutions for individual culture dishes (35 mm diameter) contained a mixture of cDNA expression vectors (1 μg for each L-type calcium channel subunits (Ca_V_α, Ca_V_β and Ca_V_α_2_δ subunits), 1 μg of AT_1_R and 100 ng of β-arrestin-RLuc) and were transfected into cells by the calcium phosphate method^27^. Experiments were conducted at room temperature 2–3 days after transfection.

### Isolation of cardiomyocytes

Rats were bred in the Animal Breeding Facility from the Facultad de Medicina, Universidad de Chile (Santiago, Chile). All studies were done in accordance and with the approval of the Universidad de Chile Institutional Bioethical Committee. Male Sprague Dawley rats (150–200 gr) were anaesthetized with ketamine plus xylazine and euthanized by isoflurane overdose before heart excision. Ventricular myocytes were isolated by enzymatic digestion in a Langendorff perfusion apparatus. Briefly, the hearts were removed, mounted on a Langendorff apparatus and perfused with modified Tyrode solution containing (mM): 133.5 NaCl, 1.2 NaH_2_PO_4_, 4 KCl, 1.8 CaCl_2_, 1.2 MgSO_4_, 10 HEPES and 5.5 glucose (pH 7.4 with NaOH) to wash out residual blood in the coronary vessels, Ca^2+^ was replaced with 5 mM EGTA after 3 min wash. Digestion was done by perfusion of the heart with modified Tyrode solution containing 25 µM Ca^2+^, 0.45 mg/ml collagenase type II (Worthington) and 0.12 mg/ml protease IV (Sigma-Aldrich) at 37 °C for 7 min, after which the Ca^2+^ concentration was increased to 50 µM for 7 min and further to 400 µM for 5 min. Subsequently, the heart was removed from the Langendorff apparatus and the dissociated cardiomyocytes were suspended in 400 µM Ca^2+^-Tyrode solution containing 1 mg/ml BSA, 30 mM taurine and 5 mM pyruvate at room temperature, and the Ca^2+^ concentration was gradually increased to 1.8 mM. Adult rat cardiomyocytes were cultured on laminin-coated coverslips (10 µg/ml) for 2 hr prior to AngII stimulation.

### Bioluminescence resonance energy transfer (BRET)

AD-293 cells were co-transfected with Ca_V_1.2-YFP and β-arrestin-Rluc fusion proteins (+Ca_V_β and Ca_V_α_2_δ) or Ca_V_β-YFP and β-arrestin-Rluc fusion proteins (+Ca_V_1.2 and Ca_V_α_2_δ). After 48 hours, cells were seeded into a 96-well microplate and coelenterazine H was added 10 minutes before AngII. Ca_V_1.2 recruitment of β-arrestin was detected every 25 seconds as the ratio of the light intensity measured at 535 ± 20 over 475 ± 20 nm using a Tecan fluorometer. BRET signal of cells expressing YFP and Rluc was compared with cells overexpressing only Rluc as control. Every BRET experiment was performed at a controlled temperature of 23 °C.

### Antibody feeding assays

AD-293 cells over-expressing hemagglutinin-epitope (HA-epitope) tagged Ca_V_1.2 (+Ca_V_β and Ca_V_α_2_δ_1_) and AT_1_R were incubated with anti-HA antibody (Roche) for 30 min at 37 °C. Subsequently, cells were stimulated with AngII (1 µM), washed with Hank’s solution and fixed with 4% paraformaldehyde. Surface-remaining Ca_V_1.2 channels were labeled with a secondary antibody (1:1000) conjugated to Alexa Fluor 488 (green, Invitrogen) in PBS. Then, cells were permeabilized with 0.5% triton in PBS (+1% BSA) and total Ca_V_1.2 channels were labeled with a secondary antibody (1:1000) conjugated to Alexa Fluor 594 (red, Invitrogen) in PBS. For image analysis, images were collected on an inverted microscope (Olympus IX-81, UPLFLN 40XO 40x/1.3 oil-immersion objective) and acquired using CellR software (Olympus). All image analyses were performed with ImageJ.

To analyze HA-epitope-tagged Ca_V_1.2 internalization, the green signal was converted to a binary image by setting the threshold function to the onset of the frequency of staining intensities histogram. With this procedure, any green pixel (independent of its original intensity) is set to maximal intensity. To obtain the percentage of internalized channels, the resultant binary image was subtracted from the red signal. This new red-signal image represents the number of internalized channels and the integrated intensities were normalized by dividing them with the integrated intensity of the red signal (that represents the total amount of channels, see Supplementary Figure 1).

### Immunofluorescence

Adult rat cardiomyocytes were seeded on laminin pretreated coverslips and then incubated with AngII (1 µM) in the presence or absence of Losartan (100 nM) (Sigma). Subsequently, cardiomyocytes were washed with PBS and fixed with 4% paraformaldehyde. Cardiomyocytes were then permeabilized with 0.02% Triton X-100 in blocking buffer (3% BSA, 5% goat serum in PBS) and incubated for 16 hours with primary antibodies (Anti-Ca_V_1.2, Alomone #ACC-003, rabbit polyclonal, Anti- βarrestin_1/2_, Santa Cruz #SC-53781, mouse monoclonal, Anti-αActinin, Abcam #AB-9465, mouse monoclonal) before addition of fluorescently labeled secondary antibodies and visualization by confocal microscopy. Intracellular signal percentage was calculated by integrating the cardiomyocyte surface fluorescence (defined by hand for each image) and subtracting it from the total integrated fluorescence. This ensuing signal was then normalized to the total integrated fluorescence.

### Colocalization analysis

Intensity Correlation Analysis, performed as described previously^29^, is based on the principle that when two proteins colocalize their staining intensities should vary synchronously^30^. Briefly, the Product of the Differences from the Mean (PDM) of each pixel was calculated and the Intensity Correlation Quotient (ICQ) was defined as the ratio of the number of positive PDM pixels to the total pixel numbers. The ICQ values are distributed between −0.5 and +0.5 by subtracting 0.5 from this ratio and a dependent staining is considered when the ICQ value range between 0 < ICQ ≤ + 0.5.

### Calcium Imaging

Plated cardiomyocytes were mounted in a perfusion chamber on the stage of an inverted microscope (Olympus IX-81, UPLFLN 40XO 40 x/1.3 oil-immersion objective). Fluorescence was collected using a CCD-based imaging system (Olympus DSU) running CellR software (Olympus). Cardiomyocytes were incubated with Fluo-4 (Molecular Probes, 1 µM) as described in ref. 28 and thoroughly washed with external solution (mM): 100 NaCl, 5 KCl, 2 CaCl_2_, 1 MgCl_2_, 90 sorbitol, 5 glucose and 10 HEPES (pH 7.4 adjusted with Tris-Base). Fluo-4 loaded cardiomyocytes were excited at 480 nm and the fluorescence emission at 510 nm was collected and recorded at 12 Hz. For each experiment, signals were recorded and the background intensity subtracted using equivalent regions of interest (ROI) outside the cardiomyocytes. Fluo-4 results are expressed as normalized fluorescence (F/F_0_).

### Electrophysiology and data analysis

Ca^2+^ currents were recorded by conventional whole-cell patch clamp^31^. Borosilicate glass pipettes were pulled to 2–4 MΩ resistance and filled with internal solution containing (mM): 108 CsCl, 4 MgCl_2_, 2 CaCl_2_, 10 EGTA 10 HEPES, 5 MgATP and 0.6 LiGTP (pH 7.2 adjusted with CsOH). The bath solution contained (mM): 5 CaCl_2_, 1 MgCl_2_, 10 HEPES, 20 TEA-Cl, 10 glucose and 100 NMDG-Cl (pH 7.4 adjusted with Tris). Data were acquired at room temperature using an Axopatch 200B amplifier and pClamp 10 software (Axon Instruments), low pass-filtered at 5 kHz, and digitized at 10 kHz. Series resistance was compensated to 85%. Data analysis, currents fitting and offline leak subtraction were performed in Clampfit 10 (Axon Instruments), and SigmaPlot 11 (Jandel Scientific). Current-voltage (IV) plots were fitted using a modified Boltzmann equation:

1 $$I=\frac{{G}_{{\rm{\max }}}\ast (V-{E}_{rev})}{1+{e}^{-(V-{V}_{a})/k}}$$

where *E*_*rev*_ is the reversal potential, G_max_ is the maximum slope conductance, *k* is the slope factor, and *V*_*a*_ the half-activation potential.

For action potential (AP) recordings, current-clamp mode was used and APs were elicited by 1 min trains of short (2 ms) depolarizing current injections at a frequency of 1 Hz, the average of the last 30 APs was used for analysis. Intracellular solution for these recordings was (mM): 120 KCl, 1.5 MgCl_2_, 0.01 CaCl_2_, 0.5 EGTA, 10 HEPES, 5 MgATP and 0.6 LiGTP (pH 7.2 adjusted with KOH). Extracellular solution was: 140 NaCl, 4 KCl, 1 MgCl_2_, 2 CaCl_2_, 10 Glucose and 10 HEPES (pH 7.4 adjusted with NaOH).

### Measurement of myocyte shortening

Contractile properties of isolated ventricular myocytes were determined by measuring the fractional shortening of single cell cardiomyocyte at room temperature and superfused with external solution (mM): 100 NaCl, 5 KCl, 2 CaCl_2_, 1 MgCl_2_, 90 sorbitol, 5 glucose and 10 HEPES (pH 7.4 adjusted with Tris-Base). Myocytes were field-stimulated with 2 ms pulses at 1 Hz. Cardiomyocyte shortening and relengthening was measured on the stage of an inverted microscope (Olympus IX-81, UPLFLN 40XO 40 x/1.3 oil-immersion objective) using a CCD-based imaging system in which the myocyte motion was collected and recorded at 12 Hz and analyzed with ImageJ.

### Statistics

Data are presented as mean ± sem (n). Statistical analysis of the data was performed with SigmaPlot 11 (Jandel Scientific) using unpaired Student’s *t*-test, and was considered significant at *P* < 0.05. One-way ANOVA test was performed for samples exposed to multiple treatments and was considered significant at *P* < 0.05.

## Results

**Figure 1:**
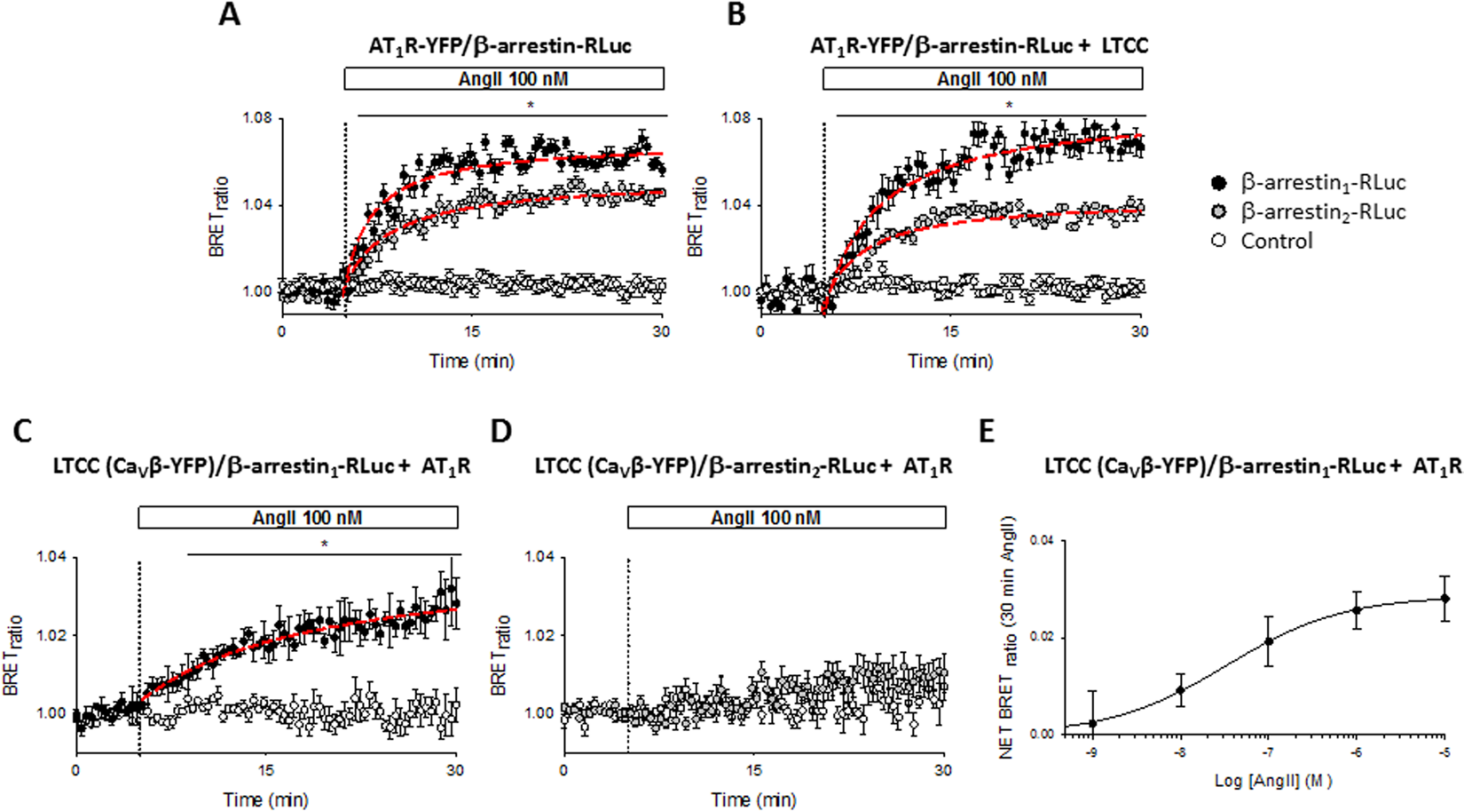
BRET assay between AT_1_R or Ca_V_1.2 channel and β-arrestins upon AngII stimulation. Time course of BRET signal ratio from AD-293 cells transfected with β-arrestin_1_-RLuc (black circles) or β-arrestin_2_-RLuc (grey circles) plus AT_1_R-YFP alone **(A)** or AT_1_R-YFP plus the L-type calcium channel (Ca_V_1.2, Ca_V_β and Ca_V_α_2_δ) **(B)**. For negative controls (empty circles), AD-293 cells were transfected without the β-arrestins. Time course of BRET signal ratio from AD-293 cells transfected with β-arrestin_1_-RLuc (**C**) or β-arrestin_2_-RLuc (**D**) plus AT_1_R and a YFP-tagged L-type calcium channel (Ca_V_1.2, Ca_V_β/YFP and Ca_V_α_2_δ). For negative controls (empty circles), AD-293 cells were transfected without the Ca_V_1.2 subunit. Red lines correspond to the best fit to a single rectangular hyperbola. The BRET records are averages of at least five independent experiments (n = 5–8). (**E**) Concentration-response curve of AngII-induced increase in net BRET signal in AD-293 cells transfected with β-arrestin_1_-RLuc plus AT_1_R and a YFP-tagged L-type calcium channel (Ca_V_1.2, Ca_V_β/YFP and Ca_V_α_2_δ), the line correspond to the best fit to Hill equation, n = 3. Mean values ± sem are shown.

In live cells, β-arrestin recruitment to activated AT_1_R can be measured by the bioluminescence energy transfer (BRET) assay^32^. AngII stimulated AD-293 cells overexpressing β-arrestin_1_/Rluc or β-arrestin_2_/Rluc together with AT_1_R/YFP display an increased BRET signal within the first minutes of stimulation (Fig. 1A). Notably, overexpression of L-type calcium channels (Ca_V_1.2, Ca_V_β and Ca_V_α_2_δ) in the same cells did not change the BRET signal (Fig. 1B). Given the fact that BRET can only occur between particles located within ~100 Å from each other, this approach enables the study of the AngII-mediated interaction between β-arrestin and Ca_V_1.2 channels in live cells. As shown in Supplementary Figure 2, a significant increase in BRET signal is observed upon AngII treatment only in those AD-293 cells expressing β-arrestin_1_/Rluc, AT_1_R and a YFP-tagged L-type calcium channel (Ca_V_1.2/YFP, Ca_V_β and Ca_V_α_2_δ), confirming the close proximity between Ca_V_1.2 complexes and activated AT_1_R. However, C-terminal fusion of YFP to the Ca_V_1.2 α subunit resulted in a relatively small BRET signal. Therefore, the experiment was modified to enhance the BRET signal by fusing YFP to the C-terminus of the Ca_V_β subunit (Fig. 1C and D), as Ca_V_β subunits has been shown to have physiological functions in the absence of Ca_V_α subunits^33^. Besides the usual negative control (cells without the YFP construct), for these experiments we used cells expressing all the constructs, except Ca_V_1.2 (β-arrestin_1_/Rluc, AT_1_R, Ca_V_β/YFP and Ca_V_α_2_δ). Under this configuration, we detected significant BRET signal changes upon AngII stimulation in those cells expressing β-arrestin_1_/Rluc, AT_1_R and the YFP-tagged LTCC channel subunits (Ca_V_1.2, Ca_V_β/YFP and Ca_V_α_2_δ). Those cells lacking in Ca_V_1.2 (Fig. 1C and D) or AT_1_R (not shown) revealed no detectable changes in BRET signal. Interestingly, we only observed an increase in BRET signal upon β-arrestin_1_ (Fig. 1C) but not with β-arrestin_2_ overexpression (Fig. 1D), even though AT_1_R activation is able to recruit both β-arrestin isoforms (Fig. 1A and B)^34^. Importantly, under our experimental conditions AngII increased the net BRET signal in a concentration-dependent manner with an apparent EC_50_ of 130 ± 50 nM (Fig. 1E, n = 3).

**Figure 2:**
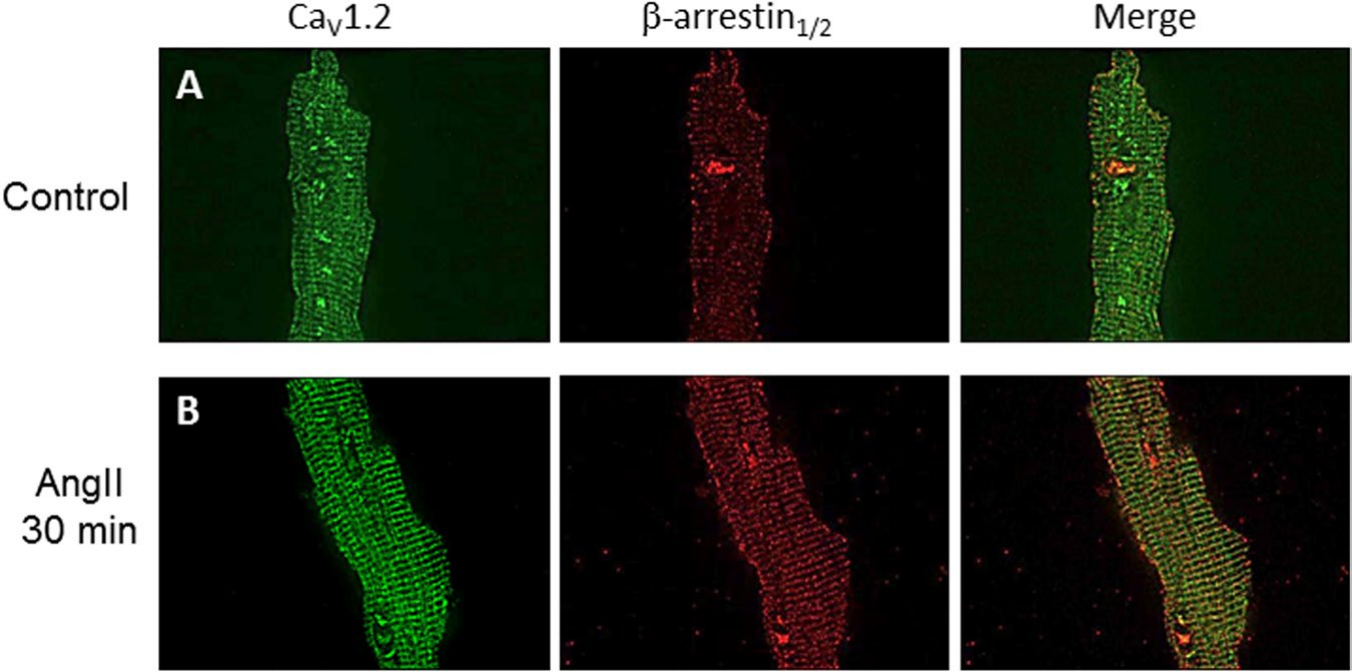
Ca_V_1.2 and β-arrestin_1/2_ immunolocalization in adult cardiomyocytes. Representative confocal images of Ca_V_1.2 (left) or β-arrestin (middle) immunofluorescences in cardiomyocytes control **(A)** or treated with AngII (1 µM) for 30 min **(B)**, the overlay of individual images is shown on the right panel. All fluorescence images were collected at the same gain setting of the microscope. The images are representatives of 6 independent experiments.

To confirm β-arrestin recruitment to the L-type channel macrocomplex in native cells, adult rat cardiomyocytes were isolated and stimulated with AngII. As expected for Ca_V_1.2 immunostaining, L-type calcium channels were observed in a regularly spaced array consistent with the arrangement pattern of T-tubules, as well as surface sarcolemmal staining (Fig. 2A and Supplementary Figure 3) which highlights different populations of LTCC in cardiomyocytes^35^. Moreover, immunostaining with a β-arrestin_1/2_ antibody revealed that in the absence of agonist, these proteins are distributed along the T-tubules and the plasmalemma with a small cluster found into the nucleus (Fig. 2A). A higher amount of β-arrestin_1/2_ into the T-tubule network was readily observed upon addition of AngII (30 min, 1 µM, Fig. 2B). To estimate the degree of colocalization of both proteins at the plasma membrane we submitted our data to intensity correlation analysis^30^, a methodology that assumes that if two proteins colocalize, their fluorescence intensities should vary synchronically above the mean fluorescence (see methods). Intensity correlation analysis revealed an increase in the mean Intensity Correlation Quotient (ICQ) values for Ca_V_1.2 and β-arrestin_1/2_ upon AngII stimulation (Control: 0.18 ± 0.05, n = 6; AngII: 0.29 ± 0.03, n = 8; p < 0.01), which suggests that β-arrestin is mobilized to the site of L-type calcium channel and thus, showing enhanced colocalization in acutely isolated rat cardiomyocytes.

**Figure 3:**
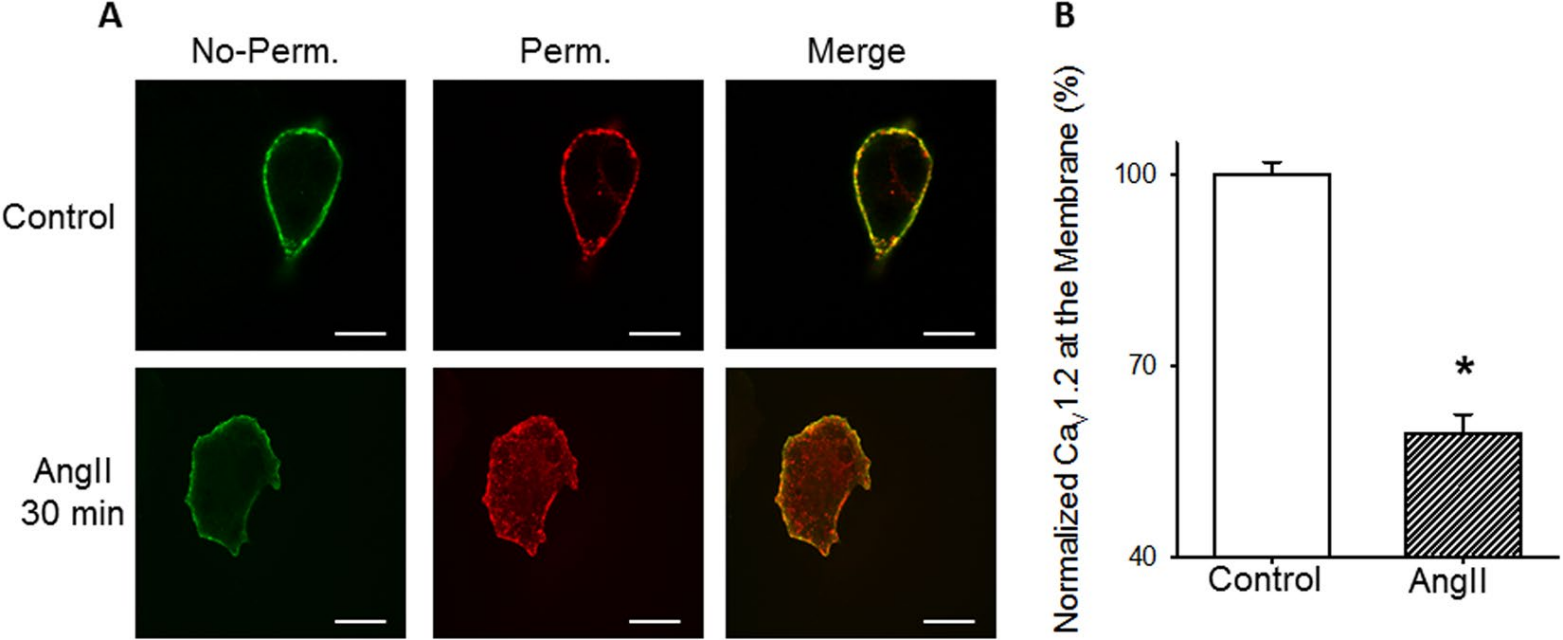
Ca_V_1.2 internalization upon AngII exposure in overexpression system. **(A)** Confocal images of the distribution of HA-epitope-tagged Ca_V_1.2 constructs in AD-293 cells over-expressing Ca_V_1.2 (plus Ca_V_β and Ca_V_α_2_δ) and AT_1_R before (top) or after (bottom) treatment with 1 µM AngII (30 min). **(B)** Summary bar graph showing the percentage of channels localized at the plasma membrane, normalized to control condition. n = 6. *p < 0.01 compared with control cells. No-perm, cells before permeabilization, representing the Ca_V_1.2 channels that remain at the plasma membrane; Perm, cell permeabilized that represent the total of channels localized at the cell surface at the beginning of the experiment.

Binding of β-arrestin to AT_1_R results not only in the desensitization of the heteromeric G-protein response, but also in the recruitment of various components of the endocytic machinery which promotes internalization of the activated receptors from the cell surface^36^. To explore if binding of β-arrestin resulted in Ca_V_1.2 internalization, we performed antibody feeding experiments (see methods) in AD-293 cells overexpressing an extracellularly tagged Ca_V_1.2 subunit (HA-Ca_V_1.2) and AT_1_R (Fig. 3). With this protocol, the channels that remained at the membrane were immunolabeled in no-permeabilized cells with a 488-green antibody, and the total of those channels initially localized at the cell surface (internalized plus those that remain at the membrane) immunolabeled with a 594-red antibody after permeabilized the cells with 0.5% triton, allowing for direct observation and relative quantification of internalized channels. Our results (Fig. 3) demonstrate a robust loss of Ca_V_1.2 signal at the plasma membrane confirming that activation of AT_1_R indeed promotes the internalization of HA-tagged Ca_V_1.2 channels.

**Figure 4:**
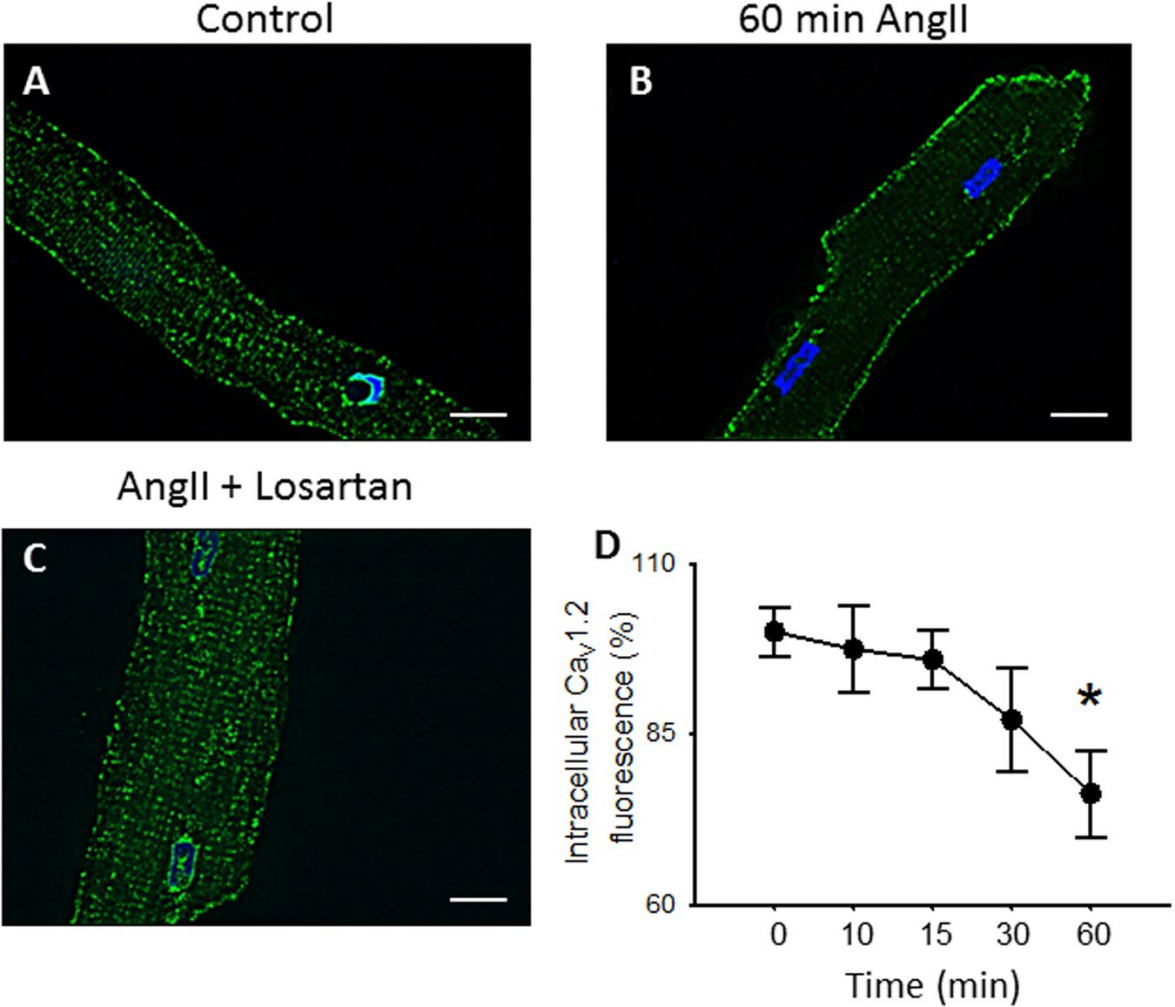
Ca_V_1.2 internalization in rat cardiomyocytes treated with AngII. Representative confocal images of Ca_V_1.2 immunofluorescence in rat cardiomyocytes, control **(A)** or treated with AngII (1 µM) for 1 hr without **(B)** or with **(C)** losartan (100 nM). All fluorescence images were collected at the same gain setting of the microscope, nucleus stained with DAPI. **(D)** Intracellular Ca_V_1.2 immunofluorescence staining intensity measurements normalized to control cardiomyocytes (n = 6–10 for each condition) from cardiomyocytes treated with AngII (1 µM) for different time points. *p < 0.01 with respect to control.

Likewise, immunostaining of Ca_V_1.2 in rat cardiomyocytes treated with AngII, at different time points, revealed a gradual removal of Ca_V_1.2 channel from the T-tubules (Supplementary Figure 3 and Fig. 4). This was significant in rat cardiomyocytes treated with AngII (1 µM) for 1 hr (Fig. 4B and D) where predominantly surface sarcolemmal staining is observed. In contrast, rat cardiomyocytes simultaneously treated with a specific AT_1_R inhibitor (Losartan, 100 nM, 1 hr) and AngII were undistinguishable from unstimulated cells (99.8 ± 5.5%, n = 4, Fig. 4C). These results suggest that only the LTCC population localized at the T-tubules is susceptible to internalization after prolonged AT_1_R activation.

**Figure 5:**
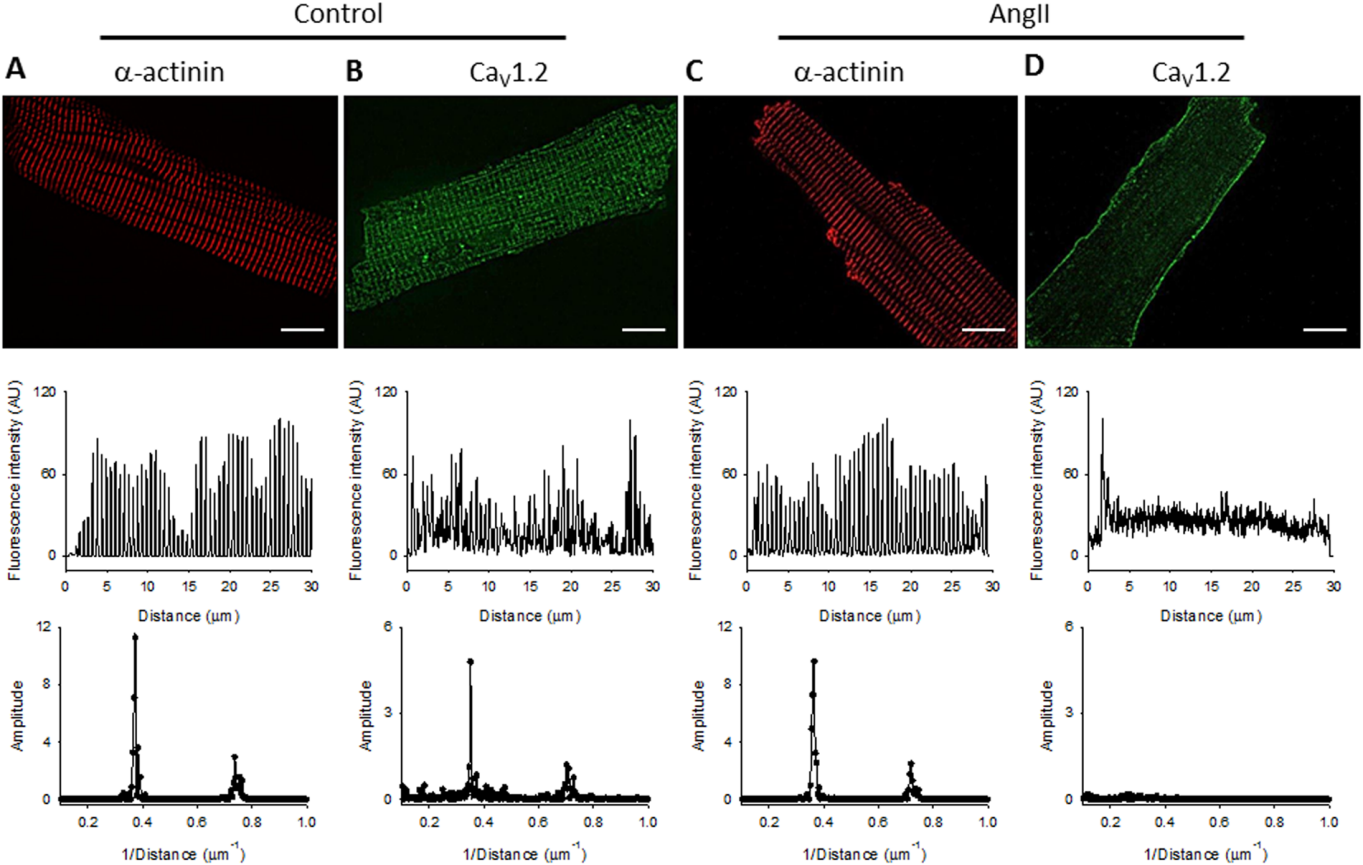
T-tubule integrity in rat cardiomyocytes treated with AngII. *Top:* representative confocal images of rat ventricular cells stained with an anti-α-actinin antibody (**A**,**C**) or an anti-Ca_V_1.2 antibody (**B**,**D**). *Middle*: Fluorescence intensity profiles, in arbitrary units, along the longitudinal axis of each cardiomyocytes. *Bottom*: graphs of the fast Fourier transformation of the fluorescence profile. **(A**,**B)** control cardiomyocytes; **(C**,**D)** cardiomyocytes treated with AngII (1 µM) for 1 hr.

Control experiments involving α-actinin staining ruled out cardiomyocyte detubulation as an explanation of Ca_V_1.2 immunostaining loss after AngII treatment. In brief, an α-actinin antibody was used (Fig. 5) and the spacing between individual T-tubules was assessed by the power spectrum of each image after a fast Fourier transform of the signal. Thus, the power spike as a function of the spatial frequency describes the average distance between individual Z-discs in the cell (Control: 2.71 ± 0.06 μm; AngII: 2.56 ± 0.11 μm, n = 5; NS), demonstrating that α-actinin immunostaining was independent of AngII treatment in our experimental conditions (Fig. 5A and C). Likewise, the average distance between Ca_V_1.2 immunostaining bands obtained in control cardiomyocytes (2.77 ± 0.03 μm, n = 5; Fig. 5B), or in Ca_V_1.2 immunostaining treated with AngII and losartan (2.67 ± 0.10 μm, n = 4; Supplementary Figure 5) were identical, in contrast, the fast Fourier transformation of Ca_V_1.2 immunostaining from cardiomyocytes treated with AngII lacks in a peak (Fig. 5D), confirming that normal spacing of L-type channels is lost in these cardiomyocytes.

**Figure 6:**
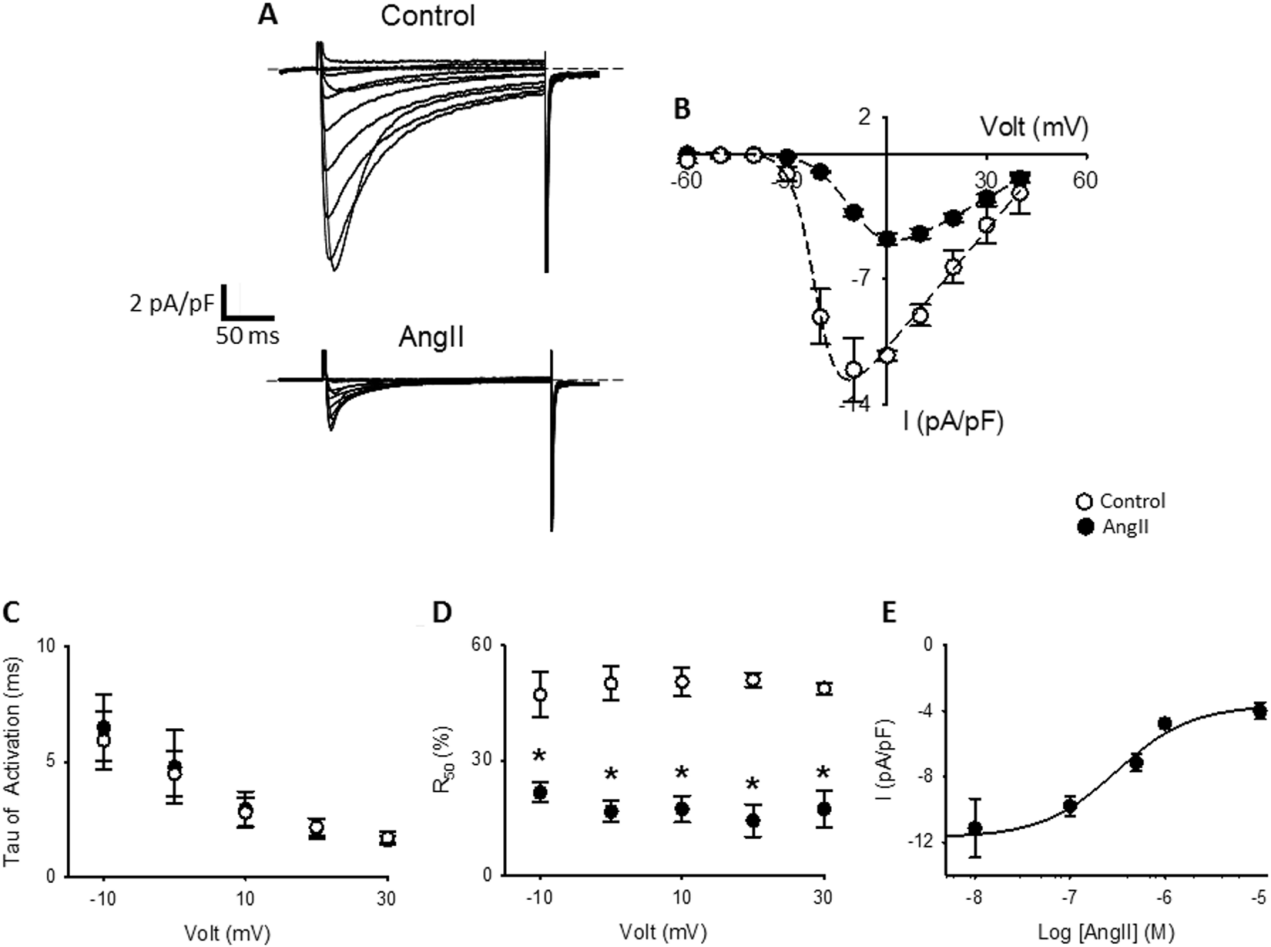
Endogenous L-type currents from cardiomyocytes treated with AngII. **(A)** Representative whole-cell endogenous L-type Ca^2+^ current traces from cardiomyocytes control (upper traces) or treated with AngII (1 µM) for 1 hr (lower traces). Currents elicited by a voltage step protocol from –60 mV to + 50 mV in 10 mV increments, V_h_ = −80 mV. **(B)** Summary peak current *I/V* plots (mean ± sem) obtained from currents family as shown in **(A)**, black line represents the best fit to a Bolztman equation (see methods). **(C)** Voltage-dependence of the activation time constant (τ_act_, mean ± sem) of L-type Ca^2+^ currents **(D)** Graph showing the residual current after 50 ms of the depolarization pulse (R_50_, mean ± sem) versus command voltage of L-type Ca^2+^ currents. Faster inactivation rates result in lower R_50_ values. **(E)** Concentration-response curve of AngII-induced L-type calcium current reduction in rat cardiomyocytes, the line correspond to the best fit to Hill equation, mean values ± sem are shown. In every panel, control rat cardiomyocytes are represented with empty circles and rat cardiomyocytes treated with AngII with filled circles. (n = 4–10) *p < 0.01 with respect to control.

To test if the AT_1_R activation has functional consequences on Ca_V_1.2, we explored the effect of AngII on endogenous L-type calcium currents in adult rat cardiomyocytes. As seen in Fig. 6A, AngII treatment (1 hr, 1 µM) induces a decrease of almost 60% of L-type calcium currents (I_max, Control_ 12.1 ± 1.8 pA/pF, n = 8; I_max, AngII_ 4.8 ± 0.4 pA/pF, n = 10; p < 0.05) and a ~10 mv right-shift in the channel voltage dependence of activation as seen in representative I-V plots (Fig. 6B). L-type current kinetics were studied by fitting a single exponential to the rising phase (activation) or by setting up the residual current after 50 ms of pulse (R_50_) at different voltages (inactivation). We did not observe differences in the activation kinetics (Fig. 6C); however, cardiomyocytes treated with AngII displayed a faster inactivation kinetics as indicated by a decreased R_50_ at all voltage tested (Fig. 6D). In agreement with our BRET experiments (Fig. 1E), a concentration dependent response, with an apparent IC_50_ of ~300 nM was observed for L-type current reduction (Fig. 6E, n = 4–10).

**Figure 7:**
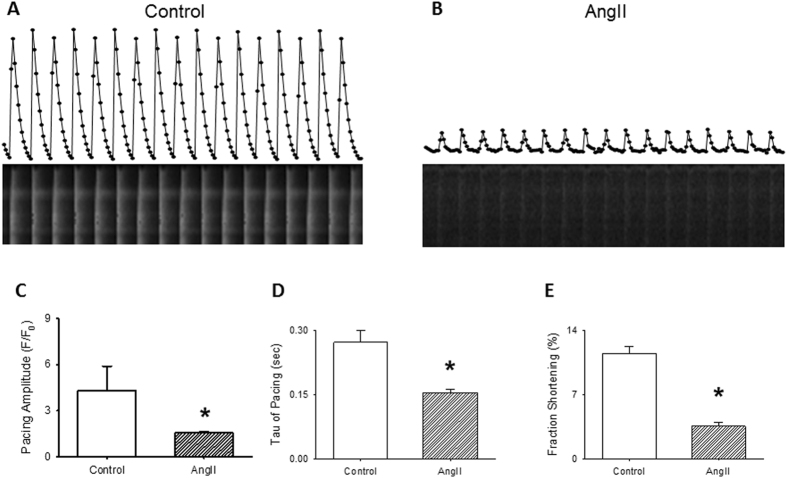
Calcium transients in cardiomyocytes treated with AngII. Representative normalized fluorescence (Fluo-4) recordings from cardiomyocytes control **(A)** or treated with AngII (1 µM) for 1 hr **(B)** and stimulated with a 5 ms external stimulus (1 Hz), individual points represent the signal for individual frames, acquire at 12 Hz. Below each fluorescence recording the respective line-scan images are shown. Bar graph (mean ± sem) of average maximal amplitude of electrically evoked Ca^2+^ transients **(C)** or time constant of Ca^2+^ decay **(D)** in cardiomyocytes control or treated with AngII (1 µM) for 1 hr. Data obtained after fitting individual calcium transients to a single exponential (n = 30–40, from 4 different cardiomyocytes preparations). *p < 0.01 with respect to control. **(E)** Bar graph (mean ± sem) of fractional shortening. For each bar graph, empty bars represents cardiomyocytes control and hatched bar cardiomyocytes treated with AngII (1 µM) for 1 hr. *p < 0.01 with respect to control.

A reduction in L-type currents is predicted to have an impact on the magnitude and/or frequency of calcium transients and consequently, on cardiomyocyte contraction. To directly investigate this, we evaluated the effect of AngII on calcium mobilization and fractional shortening. Intracellular calcium was monitored in rat cardiomyocytes loaded with Fluo-4 and paced extracellularly with a 5 ms pulse at a frequency of 1 Hz. Figure 7 illustrates representative intracellular calcium transients recorded in adult rat cardiomyocytes, in control, and treated condition (AngII 1 µM, 1 hr). As shown, calcium transients from rat cardiomyocytes exposed to AngII displayed a faster transient decay as compared to control (extracted from single exponential fits to the Ca^2+^ transient decay; Fig. 7D) suggesting that AngII also modifies SERCA activity^28^. As expected from the reduced L-type calcium currents recorded, decreased calcium transient amplitudes are evident in rat cardiomyocytes treated with AngII (63.7 ± 1.3% reduction, Fig. 7C, p < 0.05) together with slower time-to-peak. Moreover, fractional shortening assessment reveals that in rat cardiomyocytes stimulated at a frequency of 1 Hz and treated with AngII, the fractional shortening was reduced to 30.7 ± 3.7% (Fig. 7E), consistent with the observed reduction of the amplitude of calcium transients (Fig. 7C).

**Figure 8:**
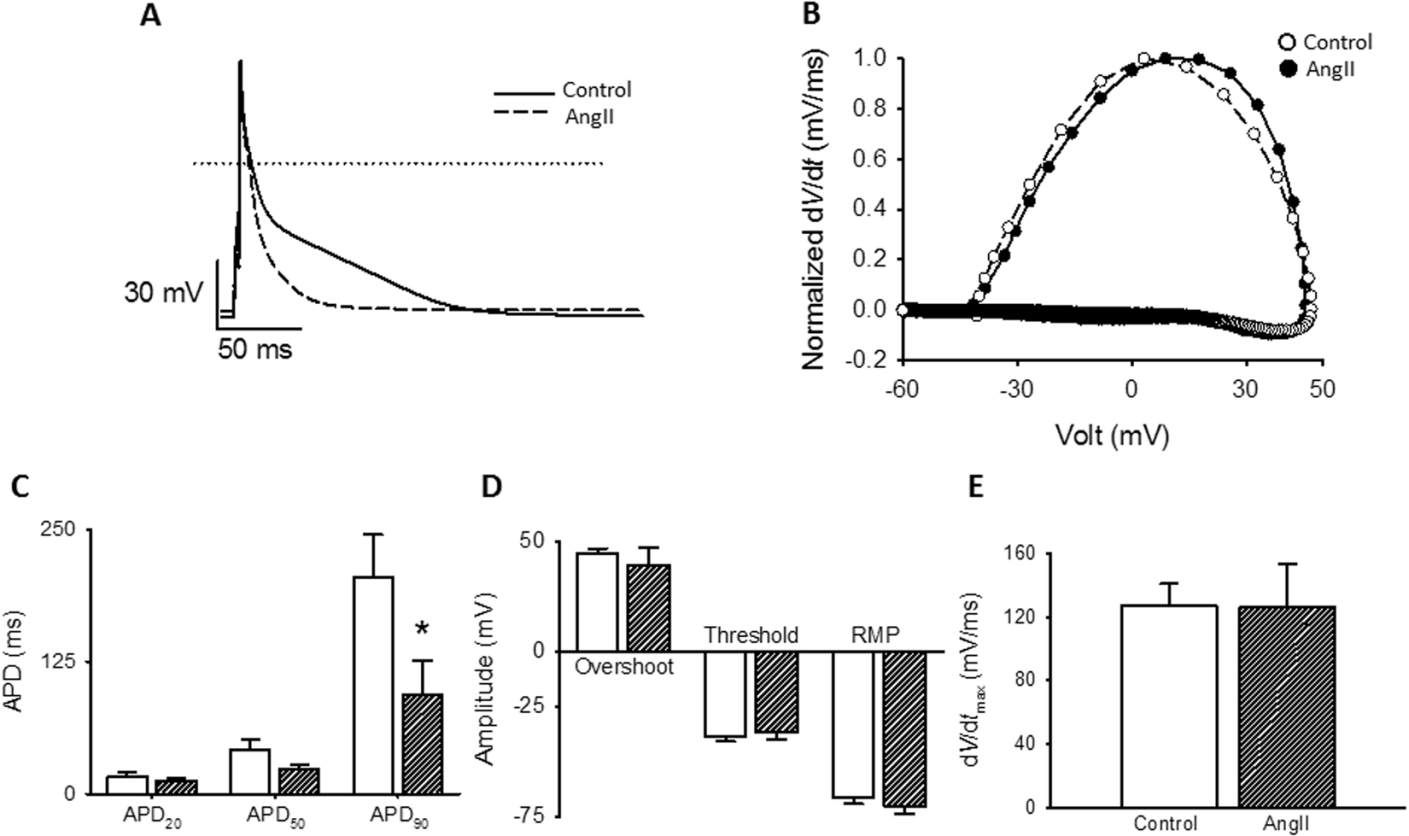
Action potentials from cardiomyocytes treated with AngII. **(A)** Representative action potentials waveforms from cardiomyocytes control (solid line) or treated with AngII (1 µM) for 1 hr (dashed line). Action potentials were elicited with a short (2 ms) depolarizing current injections (200–400 pA) at a frequency of 1 Hz. **(B)** Phase plot of the first derivative of membrane potential (d*V*/d*t*) against membrane potential (V_m_) for the action potentials shown in **(A)**. Control, empty symbols; AngII-treated, filled symbols. **(C)** Bar graphs (mean ± sem) of average action potentials duration (APD) estimated at 20%, 50% and 90% repolarization, APD_20_, APD_50_ and APD_90_, respectively. **(D)** Bar graph (mean ± sem) of maximal action potential amplitudes (Overshoot), threshold potentials and mean resting membrane potentials (RMP). **(E)** Bar graph (mean ± sem) of maximum rate of potentials change (*dV*/d*t*). For each bar graph, empty bars represents cardiomyocytes control and hatched bar cardiomyocytes treated with AngII (1 µM) for 1 hr. (n = 7–10) *p < 0.01 with respect to control.

Finally, action potentials are the result of the concerted activation of many ion channels, each of which defines distinct aspects of its shape. Careful inspection of APs confers an integrative manner to investigate putative changes in other voltage-dependent ion channels as a result of AngII prolonged stimulation. Action potential duration from ventricular cardiomyocytes was determined as the time where 20%, 50% or 90% of repolarization was attained (APD_20_, APD_50_ and APD_90_, respectively). Representative traces of superimposed APs from rat cardiomyocytes in control and after AngII treatment are displayed in Fig. 8A. Quantification of the repolarization phase shows significant differences at APD_90_ for AngII treated cardiomyocytes (Fig. 8C) suggesting that AngII treatment increases potassium currents in these cells. To examine other properties of the elicited action potentials, phase-plane plots were generated by graphing the voltage derivative in time (*d*V/*dt*) versus voltage as shown in Fig. 8B. AngII treatment does not produce significant alterations in the threshold potential, the depolarization slope or the maximal depolarization voltage. Furthermore, the resting membrane potential (RMP) remains unaltered after AngII treatment (Fig. 8D and E) which evidences lack of modification of voltage dependent sodium currents by the current experimental manipulations.

## Discussion

GPCR-ion channel macrocomplexes are thought to optimize transductional coupling by preventing the diffusion of signaling molecules and by modulating ion channel trafficking, either through regulation of channel surface expression or by mediating receptor-dependent internalization. The internalization process is slower than the activation of heteromeric G proteins activation (that usually occurs within seconds) and involves β-arrestin binding to phosphorylated receptors^37^.

The acute effects of AngII on L-type currents in cardiomyocytes have been extensively studied and appear to be species specific^20, 24^, to depend on the experimental approach used^23^ and/or on the different signaling molecules expressed^27, 38, 39^. In contrast, the prolonged effects of AngII on LTCC have received less attention. Prolonged AngII stimulation has been shown to increase the amount L-type current in newborn but not in adult mice cardiomyocytes^40^. In HL-1 myocytes, although there is a consensus that prolonged AngII-stimulation also induces an increase of L-type current, the reason for this is controversial, with a report showing a main role for β-arrestin_2_^40^ and other showing a CREB-dependent *de novo* expression of Ca_V_α_1C_^41^. Moreover, a recent study in canine cardiomyocytes suggested a dependence on the transmural muscular layer^42^, suggesting the same species-specificity as for acute AngII stimulation.

Here, we show that in cardiomyocytes from adult rat AngII increases β-arrestin_1_ recruitment to L-type Ca^2+^ channel and reduces L-type Ca^2+^ current in a concentration-dependent manner, both processes with an EC_50_ and an IC_50_ in the order of ~100 nM, respectively (Figs 1E and 6E). This AngII concentration is orders of magnitude greater than the concentration of AngII found in plasma (in the order of pM). Nevertheless, it is in good agreement with recent literature showing that prolonged high-doses of AngII produce changes at the single cardiomyocyte level^42, 43^. This difference suggests that local RAS may play a fundamental role in this process since plasma levels of AngII are too low to efficiently modify LTCC levels located at the T-tubules. In fact, recent estimates of AngII levels in canine and rat T-tubules are in the order of 100 nM^42, 44^. Thus, the local RAS at the heart^45^ could be important for electrically remodeling cardiomyocytes from different muscle layers, generating regional heterogeneities in action potential morphology and contractility^46^. Similarly, our experiments show that prolonged stimulation with AngII leads the internalization of the L-type channel population located at the T-tubules membrane (Fig. 4).

It is generally agreed that the effects of AngII in the vasculature occur at low levels of this hormone which can be used to distinguish them from the effect of AngII in the cardiac system. This can be explained by differences in receptor affinity either by post translational modification or by the formation of tissue-specific macrocomplexes involving yet unidentified protein partners. However, more work is required to establish a clearer mechanistic understanding of this process.

It should be noted that despite differences in the magnitude of the BRET signals obtained with different constructs (Fig. 1 and Supplementary Figure 2), the overall trend was consistent with a probable LTCC-β-arrestin_1_ interaction. These results are supported by immunostaining data obtained from adult rat cardiomyocytes (Fig. 2). We cannot ascertain the underlying reason for the difference in BRET signal amplitude, however some weight could be assigned to potentially different orientation of the energy transfer partners as the BRET signal depends on the orientation between donor and acceptor.

More interestingly, the BRET experiments reveal a preference for β-arrestin_1_ recruitment after AT_1_R activation as compared to β-arrestin_2_. When activated, the AT_1_R recruits both β-arrestin isoforms (Fig. 1), but ERK signaling pathway activation only occurs when β-arrestin_2_ but not β-arrestin_1_ is recruited^47^. Thus, AT_1_R interaction with Ca_V_1.2 may represent a novel form of GPCR-dependent signaling regulation in which the presence of the macromolecular complex controls the specific recruitment of β-arrestin isoforms. Understandably, this hypothesis awaits experimental corroboration.

Functionally, chronic activation of AT_1_R leads to the internalization of Ca_V_1.2 in a heterologous expression system (Fig. 3) and in ventricular rat cardiomyocytes (Fig. 4). Interestingly, internalization of Ca_V_1.2 in ventricular cardiomyocytes appears to be restricted to the population of channels present at the T-tubules known to mediate the calcium-induced calcium release process and the plateau phase of action potentials. In turn, the plasmalemma channel counterpart known to be involved in gene transcription regulation, seems unchanged by chronic stimulation with AngII. This subcellular loci-specific difference could be explained by the selective localization of AT_1_R at the T-tubules^48^ supporting the idea of the existence of an AT_1_R/Ca_V_1.2 macrocomplex in these cells.

As expected from the loss of Ca_V_1.2 signal at the T-tubule, L-type calcium currents are greatly reduced in cardiomyocytes treated with AngII (Fig. 6A). Interestingly, the remaining current display a 10 mV right-shift in the I/V curve and a faster inactivation kinetics (Fig. 6B and D), which could be a consequence either of post-translational modification of the L-type channels (as oxidation or phosphorylation due to AT_1_R activation) or, as the observed current depends mainly on the LTCC present at the plasmalemma, an indicative that the different population of LTCC between the T-tubules and the plasmalemma bears different auxiliary subunits that grant them with differential biophysical characteristics. Nevertheless, in accordance with the diminished calcium current, the calcium transient amplitudes (Fig. 7C) and cardiomyocyte shortening (Fig. 7E) are similarly decreased. Nonetheless, AngII treatment is not specific for L-type channels, as Ca^2+^-reuptake is faster in AngII-treated cardiomyocytes (Fig. 7D) likely implicating higher activity of SERCA, suggesting that AngII treatment could induce more profound changes in calcium handling than those related to mere modification of the L-type current.

Furthermore, action potential measurements (Fig. 8) allowed the monitoring of the coordinated behavior of various voltage-dependent ion channels. Despite a relatively low dependence of rat ventricular action potentials on L-type calcium currents, when compared with other species like rabbits or guinea pigs, the fact that the voltage threshold (Fig. 8D) and maximal *d*V/*dt* (Fig. 8E) were unaltered by AngII treatment clearly indicates that voltage-dependent Na^+^ channels were unaffected. Likewise, unchanged resting membrane potential in AngII-treated cardiomyocytes suggests that “leak” K^+^ channels are also unmodified. Both observations demonstrate that AngII-induced Ca_V_1.2 channel internalization does not imply an unselective effect on ion channels. In contrast, changes in APD_90_ suggest that in these tissues, a voltage-dependent K^+^-channel is activated by chronic AT_1_R stimulation which is yet to be identified.

Concerning the fate of the Ca_V_1.2 channels after prolonged AngII stimulation, those channels could be targeted either for degradation or for recycling after its internalization. In fact, a closer look of the plasmalemma-arising signal of rat cardiomyocytes treated with AngII (Figs 4 and 5 and Supplementary Figure 4) indicates a more continuous signal at the membrane edge when stained with a Ca_V_1.2 antibody, suggesting that at least part of the channels originally in the T-tubule network could be re-targeted to the plasmalemma. At the same time, as depicted from the signal loss of Ca_V_1.2 total intensity when AngII-treated cardiomyocytes are compared with control cardiomyocytes, part of the channels appear to be directed to degradation.

AT_1_R activation is well known to activate a series of intracellular signaling pathways^49^, those channels remaining at the membrane are likely to undergo posttranslational modification, such as PKC or CaMKII-dependent phosphorylation. Interestingly, these signals are usually associated with activation of the L-type current^50, 51^, suggesting that after prolonged AngII stimulation the channels in the plasmalemma, usually associated with gene transcription, are more active emphasizing the role of the process described here in cardiac remodeling.

In summary, we demonstrate a novel signaling pathway involving AT_1_R activation and LTCC. This AngII-dependent intracellular signaling pathway selectively recruits β-arrestin_1_ after receptor activation, promoting internalization of Ca_V_1.2 channels. In adult rat cardiomyocytes, the AngII driven internalization of Ca_V_1.2 occurs almost exclusively at the T-tubules leading to a significant reduction of L-type calcium current and calcium transient amplitudes. Functionally, the results presented here provide insights into the mechanisms by which prolonged AngII exposure causes cardiac remodeling and could have major implications for understanding the molecular mechanisms controlling the electrical transmural gradient observed in the heart.

## Electronic supplementary material

Supplemental Figures

## References

[1] Ruiz-Ortega M (2006). Angiotensin II: a key factor in the inflammatory and fibrotic response in kidney diseases. Nephrology, dialysis, transplantation: official publication of the European Dialysis and Transplant Association - European Renal Association.

[2] Ji TH, Grossmann M, Ji I (1998). G protein-coupled receptors. I. Diversity of receptor-ligand interactions. The Journal of biological chemistry.

[3] Miura S, Saku K, Karnik SS (2003). Molecular analysis of the structure and function of the angiotensin II type 1 receptor. Hypertension research: official journal of the Japanese Society of Hypertension.

[4] Luttrell LM (1999). Beta-arrestin-dependent formation of beta2 adrenergic receptor-Src protein kinase complexes. Science.

[5] Huang ZM, Gold JI, Koch WJ (2011). G protein-coupled receptor kinases in normal and failing myocardium. Frontiers in bioscience.

[6] Lee FJ (2002). Dual regulation of NMDA receptor functions by direct protein-protein interactions with the dopamine D1 receptor. Cell.

[7] Shukla AK (2010). Arresting a transient receptor potential (TRP) channel: beta-arrestin 1 mediates ubiquitination and functional down-regulation of TRPV4. The Journal of biological chemistry.

[8] Doronin SV, Potapova IA, Lu Z, Cohen IS (2004). Angiotensin receptor type 1 forms a complex with the transient outward potassium channel Kv4.3 and regulates its gating properties and intracellular localization. The Journal of biological chemistry.

[9] Beedle AM (2004). Agonist-independent modulation of N-type calcium channels by ORL1 receptors. Nature neuroscience.

[10] Park HW, Jung H, Choi KH, Baik JH, Rhim H (2010). Direct interaction and functional coupling between voltage-gated CaV1.3 Ca2+ channel and GABAB receptor subunit 2. FEBS letters.

[11] Kisilevsky AE (2008). D1 receptors physically interact with N-type calcium channels to regulate channel distribution and dendritic calcium entry. Neuron.

[12] Kisilevsky AE, Zamponi GW (2008). D2 dopamine receptors interact directly with N-type calcium channels and regulate channel surface expression levels. Channels.

[13] Altier C (2006). ORL1 receptor-mediated internalization of N-type calcium channels. Nature neuroscience.

[14] Simms BA, Zamponi GW (2012). Trafficking and stability of voltage-gated calcium channels. Cellular and molecular life sciences: CMLS.

[15] Best JM, Kamp TJ (2012). Different subcellular populations of L-type Ca2+ channels exhibit unique regulation and functional roles in cardiomyocytes. Journal of molecular and cellular cardiology.

[16] Beech DJ (1997). Actions of neurotransmitters and other messengers on Ca2+ channels and K+ channels in smooth muscle cells. Pharmacol Ther.

[17] Quignard JF (2001). Phosphoinositide 3-kinase gamma mediates angiotensin II-induced stimulation of L-type calcium channels in vascular myocytes. The Journal of biological chemistry.

[18] Seki T, Yokoshiki H, Sunagawa M, Nakamura M, Sperelakis N (1999). Angiotensin II stimulation of Ca2+ -channel current in vascular smooth muscle cells is inhibited by lavendustin-A and LY-294002. Pflugers Archiv: European journal of physiology.

[19] Callewaert G, Hanbauer I, Morad M (1989). Modulation of calcium channels in cardiac and neuronal cells by an endogenous peptide. Science.

[20] Ikegami H, Endoh T, Suzuki T (2001). Angiotensin II-induced inhibition of calcium currents in hamster submandibular ganglion neurons. Neuroscience research.

[21] Maturana AD (1999). Angiotensin II negatively modulates L-type calcium channels through a pertussis toxin-sensitive G protein in adrenal glomerulosa cells. The Journal of biological chemistry.

[22] De Mello WC, Monterrubio J (2004). Intracellular and extracellular angiotensin II enhance the L-type calcium current in the failing heart. Hypertension.

[23] Ichiyanagi O, Ishii K, Endoh M (2002). Angiotensin II increases L-type Ca2 + current in gramicidin D-perforated adult rabbit ventricular myocytes: comparison with conventional patch-clamp method. Pflugers Archiv: European journal of physiology.

[24] Aiello EA, Cingolani HE (2001). Angiotensin II stimulates cardiac L-type Ca(2+) current by a Ca(2+)- and protein kinase C-dependent mechanism. American journal of physiology. Heart and circulatory physiology.

[25] De Mello WC (1998). Intracellular angiotensin II regulates the inward calcium current in cardiac myocytes. Hypertension.

[26] Bkaily G (2005). Angiotensin II-induced increase of T-type Ca2+ current and decrease of L-type Ca2+ current in heart cells. Peptides.

[27] Hermosilla T (2011). L-type calcium channel beta subunit modulates angiotensin II responses in cardiomyocytes. Channels.

[28] Moreno C (2015). Cavbeta2 transcription start site variants modulate calcium handling in newborn rat cardiomyocytes. Pflugers Archiv: European journal of physiology.

[29] Altier C (2011). The Cavbeta subunit prevents RFP2-mediated ubiquitination and proteasomal degradation of L-type channels. Nature neuroscience.

[30] Li Q (2004). A syntaxin 1, Galpha(o), and N-type calcium channel complex at a presynaptic nerve terminal: analysis by quantitative immunocolocalization. The Journal of neuroscience: the official journal of the Society for Neuroscience.

[31] Varela D, Niemeyer MI, Cid LP, Sepulveda FV (2002). Effect of an N-terminus deletion on voltage-dependent gating of the ClC-2 chloride channel. The Journal of physiology.

[32] Sauliere A (2012). Deciphering biased-agonism complexity reveals a new active AT1 receptor entity. Nature chemical biology.

[33] Hofmann F, Belkacemi A, Flockerzi V (2015). Emerging Alternative Functions for the Auxiliary Subunits of the Voltage-Gated Calcium Channels. Curr Mol Pharmacol.

[34] Sanni SJ (2010). beta-Arrestin 1 and 2 stabilize the angiotensin II type I receptor in distinct high-affinity conformations. British journal of pharmacology.

[35] Shaw RM, Colecraft HM (2013). L-type calcium channel targeting and local signalling in cardiac myocytes. Cardiovascular research.

[36] Ritter SL, Hall RA (2009). Fine-tuning of GPCR activity by receptor-interacting proteins. Nature reviews. Molecular cell biology.

[37] Srivastava A, Gupta B, Gupta C, Shukla AK (2015). Emerging Functional Divergence of beta-Arrestin Isoforms in GPCR Function. Trends in endocrinology and metabolism: TEM.

[38] Weiss S, Doan T, Bernstein KE, Dascal N (2004). Modulation of cardiac Ca2+ channel by Gq-activating neurotransmitters reconstituted in Xenopus oocytes. The Journal of biological chemistry.

[39] Conforti L, Sumii K, Sperelakis N (1995). Dioctanoyl-glycerol inhibits L-type calcium current in embryonic chick cardiomyocytes independent of protein kinase C activation. Journal of molecular and cellular cardiology.

[40] Kashihara T, Nakada T, Kojima K, Takeshita T, Yamada M (2017). Angiotensin II activates CaV 1.2 Ca2+ channels through beta-arrestin2 and casein kinase 2 in mouse immature cardiomyocytes. The Journal of physiology.

[41] Tsai CT (2007). Angiotensin II increases expression of alpha1C subunit of L-type calcium channel through a reactive oxygen species and cAMP response element-binding protein-dependent pathway in HL-1 myocytes. Circ Res.

[42] Gao J (2014). Autocrine A2 in the T-system of ventricular myocytes creates transmural gradients in ion transport: a mechanism to match contraction with load?. Biophysical journal.

[43] Kim J, Gao J, Cohen IS, Mathias RT (2015). Angiotensin II Type 1 Receptor-Mediated Electrical Remodeling in Mouse Cardiac Myocytes. PloS one.

[44] Saris JJ (2002). Prorenin-induced myocyte proliferation: no role for intracellular angiotensin II. Hypertension.

[45] van Kats JP (1998). Angiotensin production by the heart: a quantitative study in pigs with the use of radiolabeled angiotensin infusions. Circulation.

[46] Clark RB, Bouchard RA, Salinas-Stefanon E, Sanchez-Chapula J, Giles WR (1993). Heterogeneity of action potential waveforms and potassium currents in rat ventricle. Cardiovascular research.

[47] Ahn S, Wei H, Garrison TR, Lefkowitz RJ (2004). Reciprocal regulation of angiotensin receptor-activated extracellular signal-regulated kinases by beta-arrestins 1 and 2. The Journal of biological chemistry.

[48] Nakayama M (2005). Chronic ventricular myocyte-specific overexpression of angiotensin II type 2 receptor results in intrinsic myocyte contractile dysfunction. American journal of physiology. Heart and circulatory physiology.

[49] Zhang P, Mende U (2011). Regulators of G-protein signaling in the heart and their potential as therapeutic targets. Circ Res.

[50] Swaminathan PD (2011). Oxidized CaMKII causes cardiac sinus node dysfunction in mice. J Clin Invest.

[51] Yang L (2009). Protein kinase C isoforms differentially phosphorylate Ca(v)1.2 alpha(1c). Biochemistry.

