# Supplementary material for: Prolonged AT1R activation induces CaV1.2 channel internalization in rat cardiomyocytes
Source: Sci Rep. 2017 Aug 31;7:10131. doi: 10.1038/s41598-017-10474-z (PMC5578992; doi:10.1038/s41598-017-10474-z)
Supplement: Supplementary file 1 — Supplemental Figures [file 41598_2017_10474_MOESM1_ESM.pdf]

# Prolonged $AT_1R$ activation induces $Ca_V1.2$ channel internalization in rat cardiomyocytes.

Tamara Hermosilla[1#], Matías Encina[1#], Danna Morales[1], Cristian Moreno[1], Carolina Conejeros[1], Hilda M. Alfaro-Valdés[1], Felipe Lagos-Meza[1], Felipe Simon[2,4], Christophe Altier[3], Diego Varela[*1]

# Supplemental Figure 1

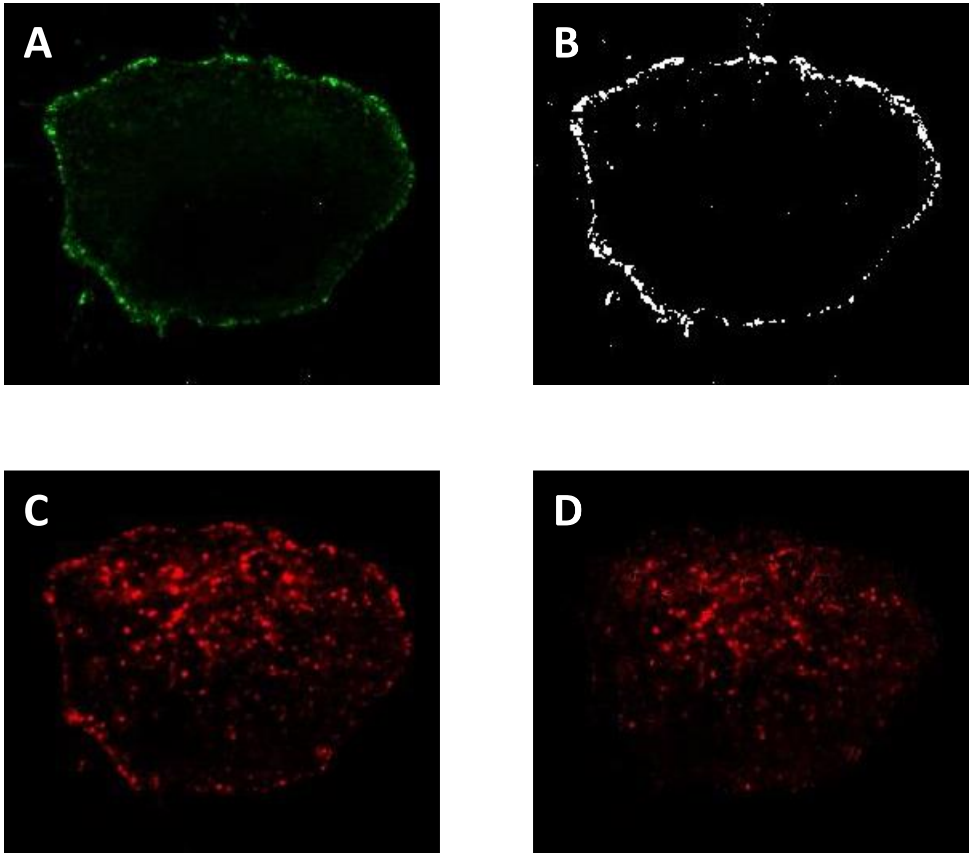


**Supplemental Figure 1: HA-$Ca_V$1.2 internalization analyzes.**
Representative confocal images of HA-$Ca_V$1.2 immunofluorescence in AD-293 cells transfected with $AT_1$R and all the subunits of $Ca_V$1.2 channel (HA-$Ca_V$1.2, $Ca_V\beta$ and $Ca_V\alpha_2\delta$) treated with AngII (1 μM) for 30 min. Cells were labeled with a 488-green antibody before permeabilization **(A)**, and with a 594-red antibody after permeabilization with 0.5% triton for 5 min **(C)**. To determine the percentage of internalized channels, the green signal was converted to a binary image **(B)** and subtracted to the red signal. The resultant image **(D)** represent the internalized channels, and was normalized with the red signal **(C)** to obtain the percentage of internalized channels.

# Supplemental Figure 2

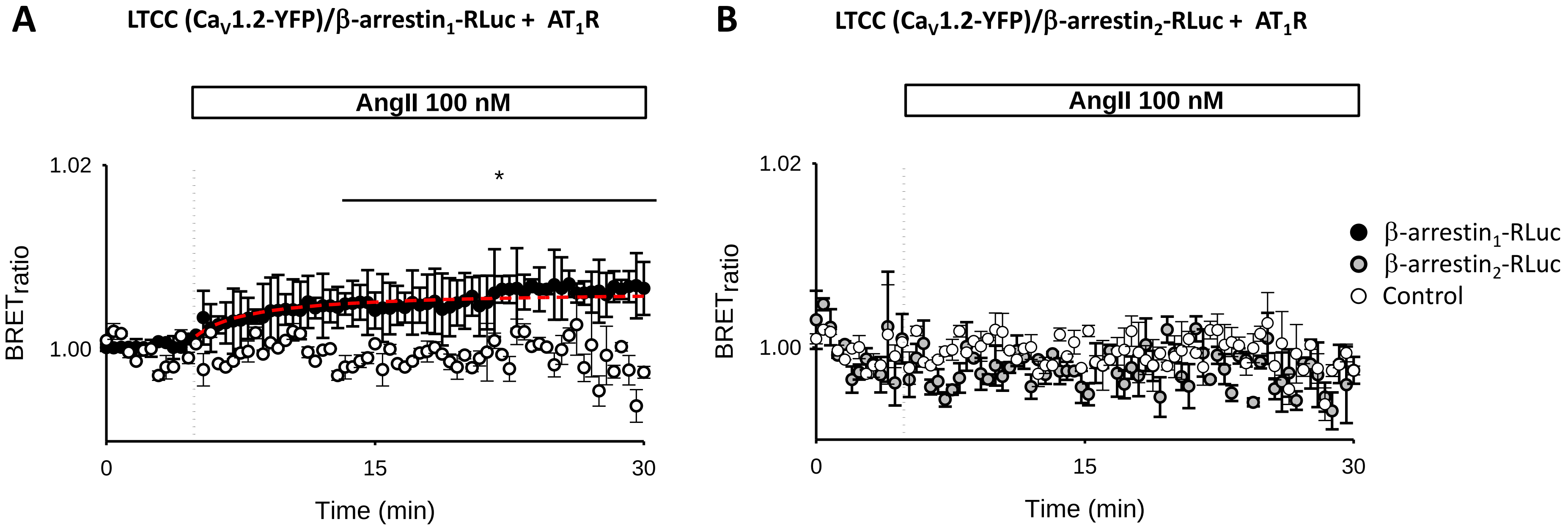


**Supplemental Figure 2:** Time course of normalized BRET signal from AD-293 cells transfected with β-arrestin$_1$-RLuc **(A)** or β-arrestin$_2$-RLuc **(B)** plus AT$_1$R and all the subunits of Ca$_V$1.2 channel (Ca$_V$1.2/YFP, Ca$_V$β and Ca$_V$α$_2$δ) (filled circles), for negative controls (empty circles) cells were transfected with Ca$_V$1.2 (without the YFP) and a membrane tagged YFP. Red lines correspond to the best fit to a single rectangular hyperbola. The BRET records are averages of at least eight independent experiments (n=8-10). Mean values ± sem are shown.

# Supplemental Figure 3

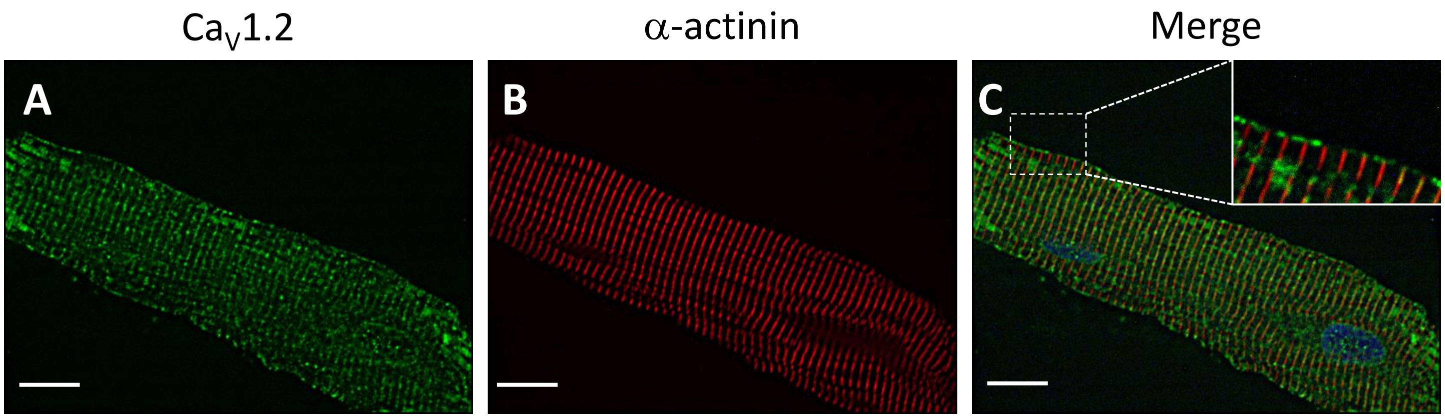


**Supplemental Figure 3: Different populations of LTCC in adult rat cardiomyocytes.**
Representative confocal images of $Ca_V1.2$ **(A)** and α-actinin **(B)** immunofluorescence in adult rat cardiomyocytes. **(C)** represent the overlaid images from **(A)** and **(B)**, nucleus were stained with DAPI. Note that the cardiomyocyte shown is the same as Figure 5A but rotated 180°.

# Supplemental Figure 4

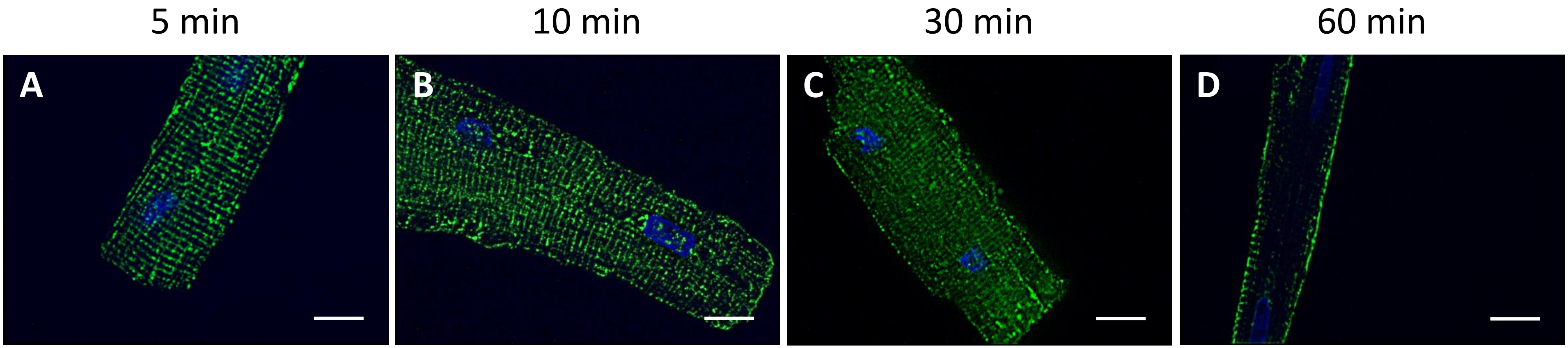


**Supplemental Figure 4: Time course of $Ca_V$1.2 internalization in rat cardiomyocytes treated with AngII.** Representative confocal images of $Ca_V$1.2 immunofluorescence in heart cells treated with AngII (1 μM) for 5 min **(A)**, 10 min **(B)**, 30 min **(C)** or 1 hr **(D)**. All fluorescence images were collected at the same gain setting of the microscope, nucleus were stained with DAPI.

# Supplemental Figure 5

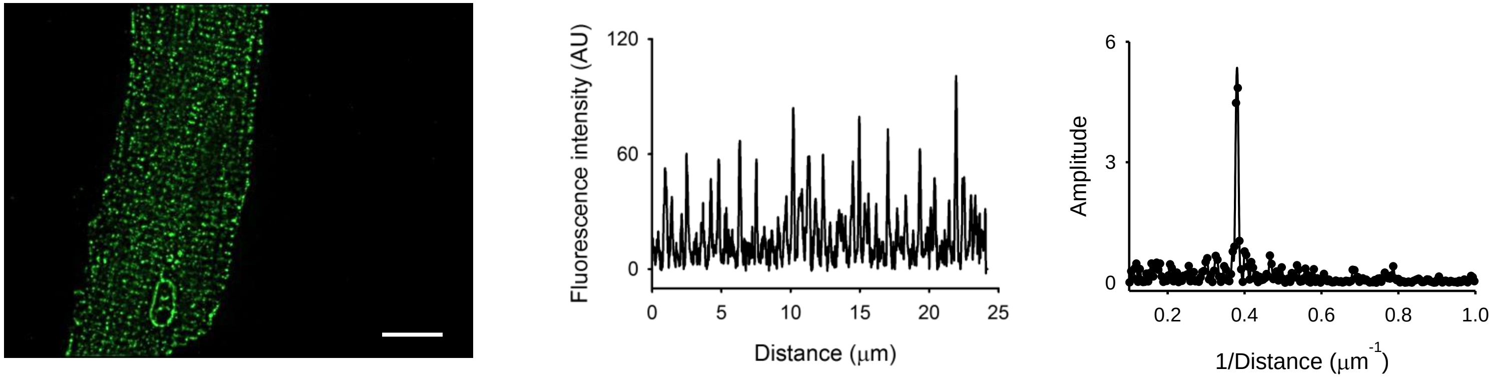


**Supplemental Figure 5: $Ca_V$1.2 distribution in cardiomyocytes treated with AngII and losartan.**
*Left:* representative confocal images of rat ventricular cells treated with AngII (1 μM) and losartan (100 nM) for 1 hr, stained with an anti-$Ca_V$1.2 antibody. *Middle*: fluorescence intensity profiles, in arbitrary units, along the longitudinal axis of the cell on the left. *Right*: graphs of the fast Fourier transformation of the fluorescence profile shown at the middle.
